# Regulatory Mechanisms of Somatostatin Expression

**DOI:** 10.3390/ijms21114170

**Published:** 2020-06-11

**Authors:** Emmanuel Ampofo, Lisa Nalbach, Michael D. Menger, Matthias W. Laschke

**Affiliations:** Institute for Clinical & Experimental Surgery, Saarland University, 66421 Homburg/Saar, Germany; lisa.nalbach@uks.eu (L.N.); michael.menger@uks.eu (M.D.M.); matthias.laschke@uks.eu (M.W.L.)

**Keywords:** somatostatin, pre-prosomatostatin, δ-cells, central nervous system (CNS), gut, hypothalamus, cAMP resonse element (CRE), pancreas/duodenum homeobox protein (PDX)1, paired box protein (PAX)6, growth hormone (GH), brain-derived neurotrophic factor (BDNF), glutamateric system, pancreas

## Abstract

Somatostatin is a peptide hormone, which most commonly is produced by endocrine cells and the central nervous system. In mammals, somatostatin originates from pre-prosomatostatin and is processed to a shorter form, i.e., somatostatin-14, and a longer form, i.e., somatostatin-28. The two peptides repress growth hormone secretion and are involved in the regulation of glucagon and insulin synthesis in the pancreas. In recent years, the processing and secretion of somatostatin have been studied intensively. However, little attention has been paid to the regulatory mechanisms that control its expression. This review provides an up-to-date overview of these mechanisms. In particular, it focuses on the role of enhancers and silencers within the promoter region as well as on the binding of modulatory transcription factors to these elements. Moreover, it addresses extracellular factors, which trigger key signaling pathways, leading to an enhanced somatostatin expression in health and disease.

## 1. Introduction

Somatostatin, also known as growth hormone-inhibiting hormone or somatotropin release-inhibiting factor, is a major product of the somatostatin gene [1]. The pre-mRNA contains an intron flanked by two exons [2]. After processing in the nucleus to produce mature mRNA, it is ordinarily degraded in the cytoplasm after several rounds of translation [3]. The biosynthesis of somatostatin is carried out via a 116-amino acid precursor protein, i.e., pre-prosomatostatin [4]. After removal of the 24-amino acid signal sequence, prosomatostatin, consisting of 92 amino acids, is formed. Prosomatostatin, in turn, is C-terminally processed to generate the cyclic peptides somatostatin-14 (SS-14) and somatostatin-28 (SS-28) [4]. Of interest, the two proteins were not identified in the same study. The isolation of SS-14 from bovine hypothalamic extracts was firstly reported in 1973 [1], while the N-terminally extended version SS-28 was described in 1980 [5]. To date, it is known that SS-14 is not only expressed in the hypothalamus but also in other parts of the central nervous system (CNS), in peripheral nerves and in pancreatic δ-cells [6]. In contrast, SS-28 is the major final product in gastrointestinal D-cells [6]. In 2008, Samson et al. [7] identified a 13-amino acid peptide, which is also encoded by the somatostatin gene. This peptide, named neuronostatin, regulates neuronal function, blood pressure, and food intake [7]. Neuronostatin is highly expressed in the spleen, pancreas, cerebrum, and hypothalamus [8].

Both SS-14 and SS-28 are stored in secretory granules and their secretion is regulated by dietary components, such as amino acids, glucose, and fat [9,10,11], as well as by the adrenergic and muscarinic systems [12,13]. Both peptides exhibit a very short half-life (~1 min) in the circulation [14]. It is estimated that ~65% of the circulating somatostatin is secreted by gastrointestinal D-cells, ~30% by the CNS and ~5% by pancreatic δ-cells [15,16,17]. The endocrine and paracrine signaling activity of somatostatin is mediated by its binding to specific somatostatin receptors (SSTRs), which belong to the class of G-protein-coupled receptors. Six different receptors (SSTR1, 2A and B, 3, 4, and 5) have been identified [18], which are widely expressed on various tissues, including retina and brain [19,20]. SS-14 binds with higher affinity to SSTRs 1-4, whereas SS-28 mainly interacts with SSTR5 [18]. After binding, SSTRs are phosphorylated, internalized into clathrin-coated vesicles, and addressed to endosomes. The receptors can then be either directly recycled to the plasma membrane or targeted by the proteasome pathway [21].

Under physiological conditions, somatostatin has a broad activity spectrum by regulating the complex balance of hormone release. For instance, it suppresses the release of growth hormone (GH), thyroid-stimulating hormone (TSH), and gastrointestinal hormones [16]. Moreover, somatostatin inhibits the secretion of insulin, glucagon, and pancreatic polypeptide from endocrine pancreatic cells [22,23] as well as the cytokine release from immune cells [24]. It also reduces the exocrine secretion of amylase of salivary glands as well as hydrochloric acid, pepsinogen, and intrinsic factor of the gastrointestinal mucosa [25,26]. Furthermore, somatostatin decreases portal pressure and retinal arteriolar and venular dilation [27,28].

SSTRs are also expressed on pathological tissues, such as neuroendocrine tumors and solid organ tumors, including melanoma, prostate, and gastrointestinal cancers [29]. The binding of somatostatin to SSTR-expressing tumor cells leads to tumor regression by reducing cell proliferation and inducing apoptosis [30]. Moreover, somatostatin is capable of indirectly suppressing tumor growth by the inhibition of angiogenesis and modulation of the immune system [31]. However, the use of native somatostatin for therapeutic approaches is limited due to its short half-life [14]. To overcome this drawback, synthetic somatostatin analogs with a longer half-life have been developed [32]. Two of them, octreotide and lanreotide, are available for the treatment of neuroendocrine tumors that secrete excessive amounts of GH [33,34].

Both the secretory mechanism and the function of somatostatin are well described [15,16,17]. However, the mechanisms regulating somatostatin expression are less well known, although the peptide hormone was discovered almost 50 years ago. Pioneering work was done by the group of Goodman [35] and Dixon [36] during the 1980s. They characterized the somatostatin promoter region and regulatory elements. In the middle of the 1990s, Montminy et al. [37] summarized the known results about the regulation of somatostatin expression. Since then, many studies have reported novel mechanisms regulating the expression of the peptide hormone, and a review about the somatostatin expression is therefore timely. In this review, we provide an up-to-date overview of these mechanisms, taking into account the gene, promoter, and transcription factors, as well as important exogenous factors.

## 2. The Somatostatin Gene

**Figure 1:**
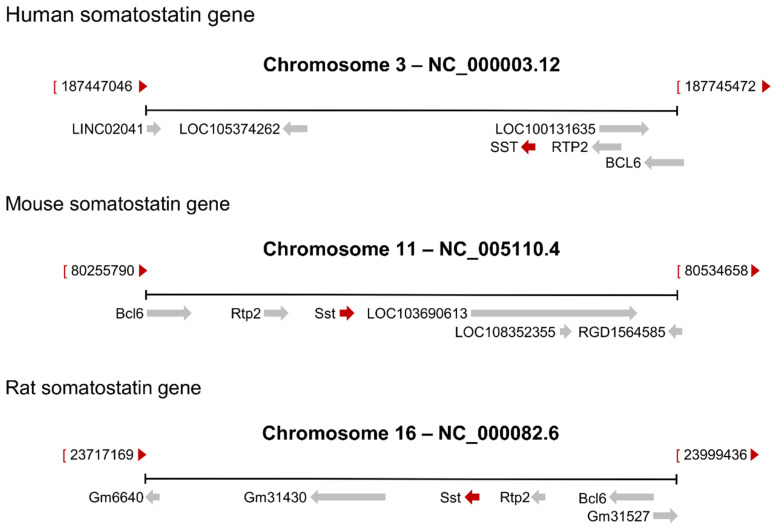
Genomic regions for the human somatostatin gene and the orthologue genes in rats and mice. Human somatostatin is located on chromosome 3, whereas mouse somatostatin is located on chromosome 11 and rat somatostatin on chromosome 16. Genomic contexts are conserved in the three species regarding the receptor transporter protein (RTP)2.

In 1982, the group of Rutter was the first to describe the sequence of the human somatostatin cDNA out of the preprosomatostatin full-length mRNA [38]. Two years later, they published the sequence of the human somatostatin gene, which is localized on chromosome 3 [2]. The ortholog gene in rats is assigned to chromosome 11 and in mice to chromosome 16. The genomic landscapes of the three orthologues are partially similar and span a region of ~1.6 kilobases (Figure 1). In the following years, it has been shown that somatostatins are a structurally diverse family of peptide hormones in vertebrates [39]. Six somatostatin genes have been identified so far [39,40]. Somatostatin 1 is expressed in all vertebrates from agnathans to mammals and represents the ancestral gene of the family [39]. Somatostatin 2, also known as cortistatin, is specifically expressed in the brain but not in the pancreas or gut [41]. Somatostatin 1, somatostatin 2, and somatostatin 5 are thought to have been produced through the 1R/2R whole-genome duplications early in vertebrate evolution [42]. Somatostatin 3 and somatostatin 6 would have been subsequently generated by tandem duplications of the somatostatin 1 as well as somatostatin 2 genes, respectively, at the base of the actinopterygian lineage [42]. In contrast, it is assumed that somatostatin 4 is derived from somatostatin 1, in teleost-specific 3R [42]. The physiological significance of somatostatin 1 is well established, whereas the different expression patterns of the other somatostatin genes might indicate that the genes have individually differing roles in various species [43]. For instance, sharks and other vertebrates show differential expression of somatostatin genes in different sets of brain neurons [44]. Gene duplication is thought to be a primary source for the evolution of novel functions [45]. In fact, it has been reported that cortistatin exerts different effects by partially antagonizing somatostatin 1 [41]. However, further studies are required to analyze whether genomic arrangements have any impact on somatostatin gene expression.

## 3. The Somatostatin Promoter

**Figure 2:**
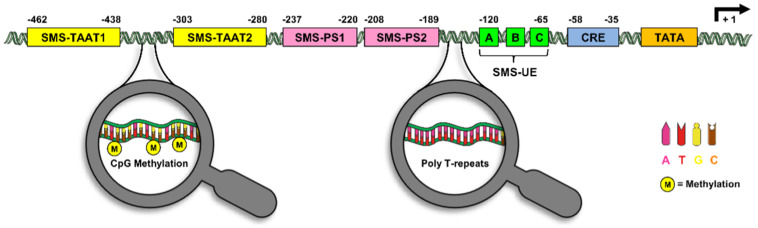
Regulatory elements of the rat somatostatin promoter. The somatostatin promoter harbors a complex arrangement of multiple regulatory elements, such as cAMP response element (CRE), specific upstream-enhancer elements (SMS-UE, SMS-TAAT1, and SMS-TAAT2) interspersed with the proximal silencer elements (SMS-PS1 and SMS-PS2) upstream of the TATA box. Moreover, additional methylation sites, i.e., GpC islets and poly-T repeats, are found in the somatostatin promoter region.

Promoters are ignition systems of genes and harbor various elements, such as enhancers and silencers, which regulate the transcriptional activation [46]. The promoter regions of the most common secretory proteins, such as insulin [47], glucagon [48], or leptin [49], are well characterized. Although somatostatin was discovered a long time ago and the mode of action of the peptide hormone is well understood, relatively little is known about the promoter region of the gene. Montminy et al. [35] were the first to identify a regulatory element in the promoter region of the rat somatostatin gene. They transfected PC12 cells with deletion mutants of a 750-bp region in 5’ to the somatostatin transcriptional start and identified a cAMP response element (CRE) with the consensus sequence ‘TGACGTCA’ [35]. This sequence resides between the nucleotides −58 and −35 upstream of the transcriptional initiation site and is crucially involved in tissue-specific somatostatin gene expression, because its mutation results in a significant loss of transcriptional activity (Figure 2) [36,50]. This is also the reason why the CRE of the somatostatin promoter is still used as a prototype to study cAMP-dependent mechanisms.

A specific upstream-enhancer element (SMS-UE), adjacent to the CRE, was first in detected in δ-cells [51]. The SMS-UE is located between the nucleotides −120 and −65 and positively regulates somatostatin gene expression synergistically with the CRE under both basal and cAMP-induced conditions [51]. Further detailed analyses revealed that SMS-UE is a tripartite element, which includes the domains A, B, and C (Figure 2) [52]. Domain A binds a ubiquitous protein with characteristics similar to the CCAAT box-binding protein CBF. Domain B harbors an insulin gene enhancer protein (Isl)-1-like binding site. Domain C contains a pancreatic islet cell enhancer sequence (PISCES) motif [52], which is found in the promoter element of the glucagon and insulin gene [53,54]. The two additional activator regions SMS-TAAT1 (−462 to −438) and SMS-TAAT2 (−303 to −280) were detected upstream of SMS-UE, which seem to be required for pancreatic somatostatin expression [55]. In contrast, Valleja et al. [56] detected the two silencer elements SMS-PS1 and SMS-PS2 resided between −250 and −120 upstream of the gene (Figure 2). These elements are not cell specific, because they are capable of reducing somatostatin gene transcription in somatostatin-producing as well as non-producing cells [56].

Beside silencer elements, the methylation of CG dinucleotides, also known as CpG islands, within promoter regions represents a common mechanism for gene inactivation [57,58,59]. In this context, it has been reported that somatostatin mRNA levels were significantly lower in the tissue of gastric cancer when compared to non-tumor tissue [60,61]. Additional analyses revealed that this is due to a somatostatin promoter hypermethylation, indicating that epigenetic modification of the promoter may be a crucial factor for gastrointestinal tract carcinogenesis [60,61]. In fact, the reduced somatostatin production due to epigenetically regulated promoter hypermethylation contributes to the uncontrolled cell proliferation in colon cancer cells, because octreotide treatment significantly attenuates cell death and cell proliferation [62]. However, whether promoter methylation also regulates physiological somatostatin expression in gastrointestinal D-cells, the CNS, or pancreatic δ-cells has still to be clarified.

Polymorphisms within promoter regions may also affect the expression of various genes and, thus, constitute common sources of phenotypic variation and susceptibility to common diseases [63]. Tremblay et al. [64] identified a poly-T repeat sequence in the somatostatin promoter ranging from 12 to 17 T. Of interest, the length of this poly-T repeat affects arterial blood pressure levels and is associated with the risk of hypertension, especially among obese individuals [64]. In a follow-up study, the authors found that the poly-T repeat polymorphism is also associated with the expression of metabolic syndrome components, indicating that this genetic alteration may induce somatostatin gene expression [65]. In fact, Li et al. [66] reported that elevated levels of somatostatin are involved in the progression of high-fat diet-induced metabolic syndrome. However, further detailed promoter analyses are required to assess the effect of polymorphisms on somatostatin gene expression.

## 4. Transcription Factors Regulating Somatostatin Expression

The expression of somatostatin is restricted to distinct tissues, indicating that cell-specific determinants control the transcription of this peptide hormone. Montminy and Bilezikjian [67] demonstrated that the transcription factor cAMP response element-binding protein (CREB) induced by the cAMP-dependent pathway binds to a promoter sequence that includes the CRE. In the following years, the binding of CREB to the consensus site TGACGTCA was verified [68,69]. Under physiological conditions, CREB is expressed in all nucleated cells and the loss of this transcription factor leads to embryonal and neuronal deficits associated with a reduced lifespan [70,71]. This clearly indicates that CREB is essential for major cellular functions. The paradigm that CREB is a crucial activator of somatostatin expression was supported by the work of Walton et al. [72]. They generated a CREB mutant (KCREB), which forms inactive heterodimers with CREB, resulting in repressed somatostatin transcription [72]. However, CREB binding does not appear to be solely responsible for the expression of somatostatin. CREB is phosphorylated by protein kinase A (PKA) on serine 133 after stimulation of the cAMP pathway [73]. This, in turn, induces the binding of the CREB-binding protein (CBP) to the transactivation domain of CREB, which further enhances somatostatin transcription [37,74]. Accordingly, the inhibition of PKA represses CREB-mediated somatostatin gene expression [75]. Beside these findings, Gachon et al. [76] detected a complex consisting of transcription activator Tax and CREB-2, also known as activating transcription factor (ATF)-4, on CRE of the somatostatin promoter. Of note, bound CREB-2 was not phosphorylated within this complex [76]. Thus, the recruitment of Tax to non-phosphorylated CREB-2 may allow the stimulation of somatostatin transcription independent of the phospho-regulated pathways.

The transcription factor pancreas/duodenum homeobox protein (PDX)1, formerly known as islet/duodenum homeobox (IDX)-1 or somatostatin transactivating factor (STF)-1, is another regulator of somatostatin gene expression. PDX1 triggers insulin gene expression in β-cells [77] and is essential for pancreas development, most probably by determining the maturation and differentiation of common pancreatic precursor cells in the developing gut [78]. In δ-cells, PDX1 is expressed at a low level [79] and activates somatostatin transcription by binding to regulatory elements in the 5′ flanking region of the rat somatostatin gene [55,80]. Further analyses revealed that PDX1 stimulates somatostatin transcription via binding to SMS-TAAT2, because mutations in this element attenuate its transactivation [55,81]. Somatostatin expression is also induced by Pbx, a transcription factor belonging to the TALE class homeobox family. Pbx is capable of forming a heterodimer with PDX1, which induces somatostatin transcription by binding to SMS-TAAT1 and SMS-UE [82]. In addition, Pbx can form heterodimers with Prep1, an additional TALE class homeobox member. However, this complex triggers somatostatin gene expression solely by binding to SMS-UE [83].

The transcription factor paired box protein (PAX) 6 is expressed in several different embryonic tissues as well as in distinct adult tissues, such as pancreatic islet cells. The complex tissue-specific PAX6-induced gene expression is made possible by several functional domains that facilitate DNA binding and protein−protein interactions [84]. It has been reported that the binding of PAX6 to the PISCES motif within endocrine gene promoters crucially regulates their gene expression [53]. For instance, Pax6 prevents the activation of insulin gene expression by occupying the PDX1 binding site in β-cells [85]. In contrast, PAX6-induced glucagon gene expression is diminished by exogenous PDX1 in α-cells [86]. The PISCES motif within the somatostatin promoter is localized in the domain C of SMS-UE [52]. Of note, PAX6 as well as PDX1 bind to completely overlapping sequences within this domain [87]. Moreover, the phosphorylation of PAX6 on serine 313 and serine 398 by extracellular signal-related kinase (ERK) is required for PAX6-mediated somatostatin transcription [74]. This indicates that beside the PKA−CREB axis, the ERK−PAX6 axis also contributes to the cell specificity of somatostatin expression.

Activin, a member of the transforming growth factor β (TGFβ) superfamily, is capable of decreasing cell proliferation in a variety of cell types [88]. The functions of activin are mediated by activin-like kinase (ALK) receptor, of which ALK4 is the main receptor mediating activin signaling in human cells [88]. Mice lacking the ALK4 receptor in GABAergic interneurons exhibit substantial deficits in medial ganglionic eminence (MGE)-derived somatostatin-expressing interneurons, which represent ~30% of all cortical GABAergic interneurons [89,90]. The development of these cells is controlled by various transcription factors, including SATB1 [91]. Recently, Göngrich et al. [89] demonstrated that SATB1 binds to different regions of the PISCES motif within the somatostatin promoter and that the activin signal alters this interaction from decreased binding to the distal region to increased binding to the more proximal region. Of note, activin does not increase somatostatin transcription, indicating that activin signaling is insufficient to regulate the expression of the peptide hormone. However, activin signaling may trigger somatostatin gene expression by reorganization of its gene locus.

## 5. Exogenous Factors Regulating Somatostatin Expression

The regulation of transcription factor activity is quite a complex process and involves post-translational modification, protein−protein interactions, as well as regulation through specific molecules, also known as ligands. These processes are mainly triggered by extracellular factors, leading to multiple intracellular signaling transductions [92]. Rage et al. [93] reported an increased somatostatin gene expression in primary hypothalamic neurons that were exposed to glutamate. In contrast, gamma-aminobutyric acid (GABA) reduces somatostatin gene expression via binding to GABA_A_ receptors [94,95]. The regulation of somatostatin expression by the GABAergic and glutamatergic system has important physiological functions, because this peptide impedes principal neurons from over-reacting by reducing their excitability and, thus, damping the rate of fire [96].

Somatostatin expression is further mediated by membrane depolarization [97,98]. It has been shown that cerebrocortical cells exposed to high K^+^ concentrations not only induce somatostatin release but also trigger its gene expression [99,100]. Of interest, this process requires the activation of Ca^2+^ channels, whereas Na^+^ channel blockade has no effect on K^+^-induced somatostatin expression [99,101]. Additional gene regulatory analyses revealed that K^+^ exposition stimulates the calmodulin/cAMP/PKA pathway, resulting in CREB-dependent somatostatin gene expression [102].

Ehrman et al. [103] detected increased somatostatin mRNA levels in islets of rainbow trout, which were cultivated under high-glucose conditions. This indicates for the first time that glucose not only regulates somatostatin release but also somatostatin biosynthesis [103,104]. In a follow up study, the authors could show that somatostatin expression is dependent on glucose-mediated hormone secretion [105]. They detected insulin-stimulated somatostatin expression only in the presence of low glucose, whereas glucagon-stimulated somatostatin expression occurred under high glucose concentrations [105]. It should be noted that the authors analyzed somatostatin 3′ and 3′’ in their study, which are not the fish counterparts of somatostatin in mammals. However, there are several indications that the expression of somatostatin seems to also be regulated by glucose in mammals. In fact, somatostatin mRNA levels were significantly increased in diabetic rats [106,107], with a return to control levels during insulin treatment [106,108]. Therefore, it is tempting to speculate that signal elements of the glucose/insulin cascades may influence CREB- or PDX1-dependent somatostatin expression.

The secretion of GH is modulated by two hypothalamic hormones, GHRH and somatostatin. Interestingly, hypoglycemia inhibits the release of GH in male rats, which is caused by a secretion of hypothalamic somatostatin [109]. Further detailed analyses revealed that insulin-induced hypoglycemia not only increased hypothalamic somatostatin release but also its gene expression in rats [110]. However, this is in contrast to the situation in humans, where hypoglycemia stimulates GH release, and the administration of glucose suppresses GH secretion [111,112]. Further studies are therefore needed to analyze whether glucose regulates hypothalamic somatostatin secretion or gene expression in humans. 

Brain-derived neurotrophic factor (BDNF) belongs to the class of neurotrophins and is highly expressed in the hypothalamus of rats [113]. BDNF signaling is primarily mediated by its affinity to the tropomyosin receptor kinase B (TrkB) [114]. TrkB activation, in turn, induces three major intracellular signaling pathways, including mitogen-activated protein kinase (MAPK), phosphatidylinositol-3-kinase (PI3K), and phospholipase C (PLC)γ [115]. Rage et al. [116] found that BNDF activates somatostatin gene expression in hypothalamic neurons, which was mediated by rapid activation of ERK1/2 and Akt kinases, resulting in the phosphorylation of CREB [117]. BDNF also enhances the activation of calcium-calmodulin-dependent kinases (CAMKs) [118]. Of interest, activated CAMK IV phosphorylates CBP at serine 301, which enhances CBP-dependent CREB transcription [119]. Hence, the suppression of CAMK activity reduces the somatostatin mRNA level [117]. Furthermore, BNDF upregulates the level of the vasoactive intestinal polypeptide (VIP), which has been shown to trigger somatostatin gene expression [120]. These findings clearly indicate that BNDF is a crucial regulator of somatostatin expression. Indeed, this factor significantly controls neuronal survival at the early stages of brain development by inducing somatostatin gene expression via different signaling pathways.

## 6. Putative Autocrine Feedback

Autocrine feedback loops are mechanisms that allow cytokines or hormones to modulate the mode of action of their own cell. Although these mechanisms are not widespread, some have already been identified in detail [121,122,123]. For instance, autocrine GH increases cell survival, proliferation, and motility, as well as decreases cell apoptosis in GH-secreting breast adenocarcinoma cells [124]. Glucagon, secreted by pancreatic α-cells, even upregulates its own gene expression by binding to the glucagon receptor, which leads to activated PKA-dependent signaling pathways [125]. It is not known whether somatostatin is also able to regulate its own expression, but there are some indications that this might be possible. The binding of somatostatin to SSTR5 mediates an inhibitory effect on islet cell survival and insulin expression [126,127]. Zhou et al. [128] reported that SSTR5-induced signaling is linked to PDX1. They found, that treatment of insulinoma cells with a SSTR5 agonist reduces glucose-stimulated insulin secretion due to diminished PDX1 expression. Of interest, PDX1 has been shown to increase somatostatin expression [55,81]. This is in line with recent findings demonstrating that antagonizing SSTR5 increases glucagon like peptide-1 as well as somatostatin secretion from the perfused proximal small intestine in mice [129]. Therefore, it is tempting to speculate that somatostatin is capable of regulating its expression by a negative feedback loop via SSTR5. 

Adenylyl cyclase (AC) catalyzes the conversion of ATP to cAMP, which, in turn, activates PKA [130]. It has been reported that SSTR signaling pathways efficiently inhibit AC via coupling to G_i_ proteins [131]. This leads to a decreased cellular cAMP level, which reduces pituitary hormone secretion and may also contribute to the antiproliferative effects of somatostatin [132,133,134]. The expression of somatostatin itself is, as already stated, induced by cAMP via the PKA−CREB-axis, hence reduced AC activity after SSTR activation may repress somatostatin expression. However, detailed studies are now required to determine whether somatostatin affects its own expression.

## 7. Conclusions

**Figure 3:**
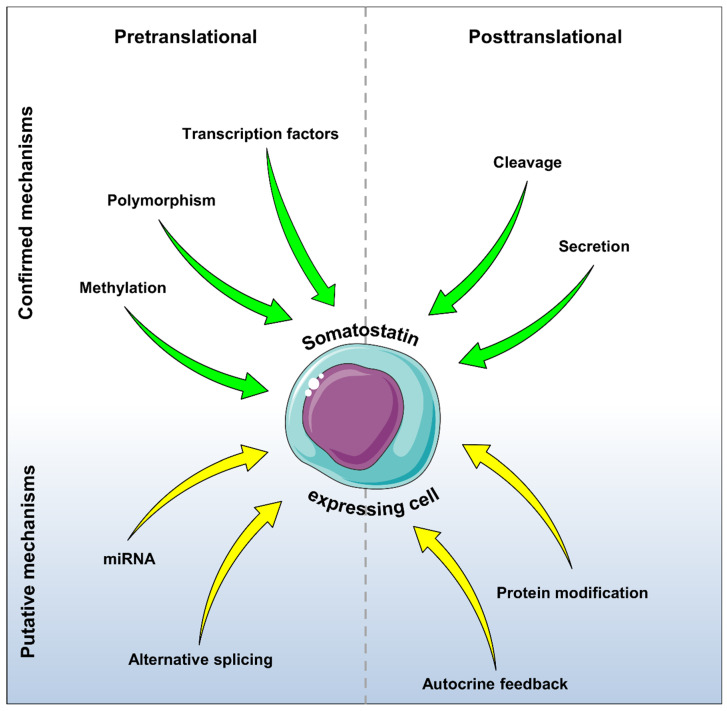
The regulatory mechanisms of somatostatin expression. It is known that the cellular somatostatin content is pretranslationally regulated by methylations and polymorphisms within the promoter region as well as by the activity of different transcription factors (green arrows). On the posttranslational level, the cellular somatostatin content is regulated by proteolytic cleavage of pre-prosomatostatin into somatostatin (SS-14 and SS-28) and by secretion (green arrows). Further putative factors and mechanisms, which may regulate the expression of the peptide hormone, are miRNAs, alternative splicing, autocrine feedback, and protein modification (yellow arrows).

The understanding of the regulatory mechanisms of somatostatin expression has markedly increased over the past decades. In addition to the known post-translational mechanisms, i.e., proteolytic cleavage of pre-prosomatostatin and somatostatin secretion, it has been shown that different pretranslational mechanisms, such as modifications of the promoter by methylations and polymorphisms as well as the regulation of transcription factor activity, are required for regulation of the cellular somatostatin content (Figure 3). Future studies now have to clarify whether additional factors and mechanisms are involved in the regulation of somatostatin expression. These may include miRNAs, which play an important role in the fine-tuning of protein expression [135], the process of alternative splicing [136] as well as post-translational protein modifications, such as phosphorylation and sumoylation [137] (Figure 3).
